# Impact of Mézières Rehabilitative Method in Patients with Parkinson's Disease: A Randomized Controlled Trial

**DOI:** 10.1155/2017/2762987

**Published:** 2017-12-03

**Authors:** Teresa Paolucci, Federico Zangrando, Giulia Piccinini, Laura Deidda, Rossella Basile, Enrico Bruno, Emigen Buzi, Alice Mannocci, Franca Tirinelli, Shalom Haggiag, Ludovico Lispi, Ciro Villani, Vincenzo M. Saraceni

**Affiliations:** ^1^Complex Unit of Physical Medicine and Rehabilitation, Policlinico Umberto I Hospital, “Sapienza” University of Rome, Piazzale Aldo Moro 5, 00185 Rome, Italy; ^2^Department of Physical Medicine and Rehabilitation, San Camillo-Forlanini Hospital, Circonvallazione Gianicolense 87, 00151 Rome, Italy; ^3^Department of Public Health and Infectious Diseases, “Sapienza” University of Rome, Piazzale Aldo Moro 5, 00185 Rome, Italy; ^4^Department of Physical Medicine and Rehabilitation, A.C.I.S.M.O.M., San Giovanni Battista Hospital, Via Luigi Ercole Morselli 13, 00148 Rome, Italy; ^5^Department of Neurology, San Camillo-Forlanini Hospital, Circonvallazione Gianicolense 87, 00151 Rome, Italy; ^6^University Department of Anatomic, Histologic, Forensic and Locomotor Apparatus Sciences, Section of Locomotor Apparatus Sciences, Policlinico Umberto I Hospital, “Sapienza” University of Rome, Piazzale Aldo Moro 5, 00185 Rome, Italy

## Abstract

The aim of this study was to assess the efficacy of Mézières method in improving trunk flexibility of the back muscles and balance in patients with Parkinson's disease (PD).* Materials and Methods*. Thirty-six patients were randomized into 2 groups: the Mézières treatment group and the control group (home exercise group). The primary outcome was the improvement in balance per the Berg Balance Scale (BBS) and the trunk flexibility of the back for the anterior flexion trunk test. Also, we evaluated pain, gait balance for the Functional Gait Assessment (FGA), disease-related disability for the Modified Parkinson's Activity Scale and the Unified Parkinson's Disease Rating Scale (UPDRS), the quality of life, and the functional exercise capacity. All the measures were evaluated at baseline (*T*0), at the end of the rehabilitative program (*T*1), and at the 12-week follow-up (*T*2).* Results*. In the Mézières group, the BBS (*p* < .001) and trunk flexion test (*p* < .001) improved significantly at *T*1 and remained the same at *T*2. Between groups, significant changes were reported in FGA (*p* = .027) and UPDRS Total (*p* = .007) at *T*1 and in FGA (*p* = .03) at *T*2.* Conclusion*. The Mézières approach is efficacious in improving the flexibility of the trunk and balance in PD patients.

## 1. Introduction

The Mézières method was created and is used to restore global mobility of joints and muscles, allowing posture reharmonizing, particularly by changing the alignment of the curves of the spine in the sagittal plane [1–3].

In Parkinson's disease, a tendency to bend or flex forward is the most common change in posture linked to a shortening of the muscular back kinetic chain [4].

It is not known why this occurs, but it may be due to many factors including muscle rigidity, brain changes that control posture, or dystonia. Muscle rigidity and imbalance of bigger muscles overpowering the smaller muscles can cause the patient to bend over [5].

Also, patients with PD usually present with impairments in motor control and sensory integration, causing static and dynamic postural control deficits: balance and gait limitations are not fully addressed by pharmacological agents in PD necessitating a nonpharmacological approach as rehabilitation. The existence of a biased representation of verticality in PD, resulting in severe retropulsion and recurrent falls, has prompted interest in a novel rehabilitation method that is dedicated to the sense of verticality [6, 7]. Most conventional and innovative exercises in PD are focused on the motor features of posture and gait, ignoring the perceptive aspects of balance. Introducing perceptive training to the exercises that are proposed for patients with PD is necessary to reduce their static and dynamic balance limitations and increase the efficacy of rehabilitative programs [8].

PD patients have to rethink their individual motor and cognitive resources to perceive, which is highly challenging in maintaining balance; thus, balance training needs to be specific and progressive [9]. Also, patients with PD have greater postural sway versus healthy subjects, which is significantly associated with a major risk of falls [10].

There is limited evidence about the efficacy of a specific physiotherapy treatment program over another in improving balance in PD. For example, there is weak evidence that freely coordinated resistance training is more effective than balance training [11], whereas complementary physical therapies, such as dancing and martial arts, hydrotherapy, virtual reality and exergaming, motor imagery, action observation, and robotic gait training, appear to have therapeutic benefits, increasing mobility and quality of life in certain patients with PD [12].

A rehabilitative program for PD should be “goal-based” (targeted towards practicing and learning specific activities), but several practice variables (intensity, specificity, and complexity) must be identified, and the program should be tailored to individual patient's characteristics [13].

On this basis, between various postural rehabilitation approaches, the Mézières method [1–3] embodies the characteristics that are useful for balance rehabilitation in patients with PD: establishing alignment according to a vertical reference and reminding the patient of motor imagery in perceiving and imagining body posture. Mézières's concept is a radical shift in therapeutic approaches, valuing relaxation, tonic inhibition, and global and progressive stretching of the muscular regions with imbalances [1–3].

Also, one of the most common nonmotor symptoms of PD is chronic pain. Pain perception is altered in PD, for example, manifesting as elevations in sensory threshold, wherein the interaction between sensory input and motor output modulates pain perception [14]. In particular, lower limb pain is a variant of central pain and merits recognition as a specific nonmotor phenotype in PD [15]. Mézières physiotherapy is effective in other chronic pain conditions, such as low back pain [16], like other muscle stretching programs, such as the Global Postural Reeducation (GPR) approach; both rehabilitation methods have various levels of progression and advocate stretching the antigravity muscle chains with parallel enhancement of the basal tone of antagonistic muscles to improve static and dynamic stability [16]. To date, there is no published article evaluating the Mézières method alone for people with PD. A few studies utilized this technique as part of a rehabilitation program for individuals with PD [17, 18].

Thus, the aim of this research is to determine the efficacy of the Mézières method in trunk flexibility of the back muscles and balance in patients with Parkinson's disease (PD).

## 2. Materials and Methods

### 2.1. Design

We conducted a single-blinded, randomized, controlled trial with a 3-month follow-up to determine the efficacy of a rehabilitative protocol, based on the Mézières method, with regard to balance and posture in patients with PD.

Patients of either gender who had been diagnosed with idiopathic PD for at least 1 year were enrolled from the physical medicine and rehabilitation outpatient clinic of Policlinico Umberto I Hospital, Sapienza University of Rome (Italy), and the neurological outpatient clinics of S. Camillo-Forlanini Hospital and S. Giovanni Battista Hospital of Rome (Italy) from July 2015 to January 2016. Eligible patients were referred to a physiatrist who was uninvolved in the study, who provided them with detailed information on the experimental protocol and performed a standardized, blinded assessment at baseline and at the follow-up to minimize potential bias when performing the clinical examination and recording the data. To maintain the blinding and limit the risk of biased observations, the examiner did not have access to the clinical examination results.

To ensure that participants were assessed under similar conditions during each examination session, all procedures were completed within 1-2 h after the patients took their medications, allowing the participants to feel comfortable and safe during the examination and the results to be representative of how a subject performed a similar task in everyday life. All tests were performed during the “on” phase.

Forty-six patients were screened, 36 of whom were enrolled and randomized into 2 groups: the Mézières treatment group (MTG: *N* = 17, median age 66.00 and IQR 18.50) and the home exercise group, or control group (CG: *N* = 17, median age 67.00 and IQR 11.00). A statistician provided a computer-generated randomization list at a ratio of 1 : 1 (MATLAB R2007b®, MathWorks Inc., USA). Sealed envelopes were prepared for each group. Participants received their randomization letter after the first neurological visit was completed.

### 2.2. Participants

Patients were recruited after a neurological examination and then subjected to a physiatrist visit. The inclusion criteria were a diagnosis of idiopathic PD with a level on the Hoehn and Yahr scale ≤ 3 (in the “on” phase) [19], age between 40 and 80 years, Mini-Mental State Examination score > or = 27 [20, 21], other disabling diseases that affected movement and gait, and steady pharmacological treatment with anti-Parkinson agents for at least 1 month.

The exclusion criteria were cognitive and visual impairments that could prevent the understanding and execution of the tasks; engagement in other rehabilitative study protocols; participation in a conflicting research study; previous treatment with deep-brain stimulation for symptom management; significant neck, shoulder, or back injuries; and uncontrolled hypertension or heart failure, rheumatic diseases, tumors, and other neurological pathologies associated.

This study was approved by the ethical committee of Sapienza University of Rome (registration number 2519/15, ClinicalTrials.gov identifier NCT02891473). All participants signed informed consent forms after receiving detailed information about the study's aims and procedures as per the Declaration of Helsinki. The rights of human subjects involved in the study were protected. This study protocol was developed in accordance with the Consolidated Standards of Reporting Trials (CONSORT) guidelines [22].

### 2.3. Measures

Sociodemographic and clinical data were collected at baseline. The following outcome measures were assessed at baseline, 1 day before starting the treatment (*T*0), at the end of the rehabilitative program (10 sessions for 5 weeks) (*T*1), and at the 12-week follow-up (*T*2).

The primary outcome was the improvement in balance per the Berg Balance Scale (BBS). The BBS is a widely used instrument that measures static and dynamic balance by assessing performance on functional tasks. It includes a series of 14 simple tasks, each of which is scored from 0 (lowest level of function) to 4 (highest level of function). The maximum score is 56 (41–56 = low risk of falls; 21–40 = medium risk; 0–20 = high risk) [23].

Pain was evaluated using the Visual Analog Scale (VAS). The VAS is a simple and sensitive instrument that enables patients to express their pain intensity as a numerical value. Patients were asked to mark the point that corresponded to their perceived pain intensity on a 10 cm line (0 = absence of pain, 10 = most severe pain) [24].

Balance and posture were evaluated using the Functional Gait Assessment (FGA) scale, which measures walking balance activity and was developed from the Dynamic Gait Index (DGI) to improve reliability and decrease the ceiling effect. The FGA consists of 10 items, each of which is scored on a 3-point scale from 0 (severe impairment) to 3 (normal deambulation). The highest possible score is 30 (normal gait function) [25].

Parkinson's disease symptoms and disease-related disability were recorded using the Modified Parkinson's Activity Scale (MPAS) and Unified Parkinson's Disease Rating Scale (UPDRS). The MPAS comprises 14 items in 3 domains: chair transfer, gait akinesia, and bed mobility. Scores range from 0 (dependent) to 4 (normal), and the highest possible score is 56 [26, 27].

The UPDRS is the most commonly used scale for monitoring the course of the disease in PD patients. It consists of 6 parts, with questions on mental state, behavior and mood, ADL, motor functions, complications of advanced disease, stage of disease per the Hoehn and Yahr scale, and abilities in everyday life activities per the Schwab and England scale. The UPDRS is based on a metric scale, ranging from 0 (no disability) to 147 (severe disability) points [28].

In this study, we measured scores for UPDRS Part I: Mentation, Behaviour, and Mood; UPDRS Part II: Activities in Daily Living; UPDRS Part III: Motor Examination; and UPDRS Part IV: Complications of Therapy and Total.

Trunk flexibility was analyzed by evaluating the anterior flexion of the trunk, measuring the finger-to-floor distance.

Functional exercise capacity was measured through the six-minute walking test (SMWT). It is a practical simple test that measures the distance that a patient can quickly walk on a flat, hard surface in a period of 6 minutes. The individual is requested to walk as far as possible in six minutes [29].

The Short Form 36 Health Survey (SF-36) was administered to collect information about health status and quality of life. The SF-36 is a generic multidimensional health questionnaire that collects practical, reliable, and valid information about patients' functional health and well-being [26]. It comprises 36 items and two overall indices that summarize the physical and mental health. Physical health includes 4 domains: physical functioning (PF), physical role functioning (PR), bodily pain (BP), and general health perceptions (GH). Emotional health, instead, includes the domains of mental health (MH), social role functioning (SF), emotional role functioning (RE), and vitality (VT). Each scale ranges from 0 to 100 (worst and best health state, resp.). The questionnaire has already been validated in Italian [30, 31].

### 2.4. Rehabilitative Intervention

For both groups, the treatment program aimed to prevent and reduce pain; the exercise was not performed if it increased pain or put the patient's safety at risk. In both programs, the level of the exercises could be adjusted step by step, based on the observations of the physician and physical therapist and the patient's needs.

#### 2.4.1. Mézières Treatment Group

The Mézières treatment regimen consisted of 3 postures that could be adapted to each patient, depending on his/her needs to correct variations in the dorsal curve and promote diaphragmatic breathing. The first objective was to recover extensibility of the hypertonic muscle groups and, in particular, those in the low back muscular chain: the paravertebral muscles and latissimus dorsi.

During execution of the postures, the physiotherapist always required the patient to follow the rhythm of his breathing, perceive the alignment of his trunk, and imagine the posture that was instructed before executing it (to promote motor imagery and attention with respect to the movement) to constantly raise awareness of the posture that was requested. All postures obligatorily passed the alignment in the same plane of 3 levels: the occipital bone, the scapula (7th thoracic vertebra), and the sacrum. Each session comprised a sequence of postures that were held to maintain rigorous and prolonged tension of the muscle groups that were considered to be responsible for the lordosis, internal rotations, and inspiratory thoracic block.

The treatment was administered over sessions twice per week for 5 weeks. Each session lasted for 1 hour and was performed by a trained physical therapist on this method. The Mézières method is usually performed for 1 or 2 sessions per week in adults, with often more than 60 minutes per session, and always twice for children. In this study, we administered 2 sessions per week, also considering that the rehabilitation guidelines for PD recommend more than one session per week [32, 33].

*First Posture*. The patient was placed in the supine position and aligned, based on his vertical line (occipital bone, 7th dorsal vertebra, and sacrum), to recreate the correct curves according to the lordosis of the spine. Then, the patient was asked to first breathe normally and then perform diaphragmatic breathing, focusing on the use of the rectus abdominis to lower the last thoracic ribs. 

*Second Posture*. The patient was placed in the supine position, with the upper limbs abducted to 120° (to obtain maximum elongation of the latissimus dorsi). This posture aims to achieve bilateral passive stretching of the latissimus dorsi. The patient was also requested to change this position by performing isometric contraction of the latissimus dorsi in the maximum elongation permitted. The physical therapist corrected the patient's raising of the last thoracic ribs on expiration. 

*Third Posture*. The patient was placed in the supine position, with the lower extremities elevated at more than 90° of flexion of the hips and the knees extended or flexed, resting on a wall or supported by the physiotherapist, if the patient was unable to reach this position with the knees extended. This exercise aimed to stretch the posterior muscle chain and especially the latissimus dorsi. Extreme care was taken to prevent inspiratory block.

**Figure 1:**
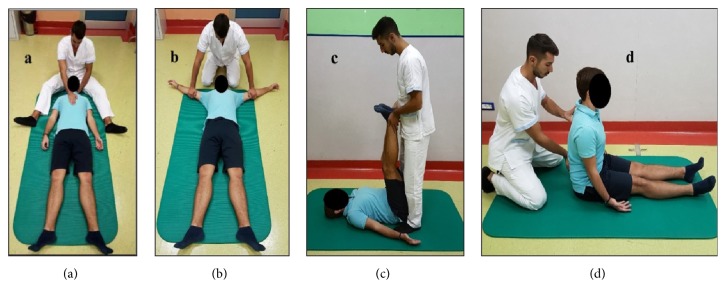
Mézières rehabilitative method: (a) the first posture, (b) the second posture, (c) the third posture, and (d) variation of the third posture. Source of pictures: UOC Physical Medicine and Rehabilitation Unit, Policlinico Umberto I Hospital, Rome, Italy.

The patient was sitting with his back leaning against the floor and aligned to his vertical line (occipital bone, 7th dorsal vertebra, and sacrum) to recreate the correct curves according to the lordosis of the spine. Then, the patient was asked to perform normal breathing and then diaphragmatic breathing to activate the rectus femoris (physiological position of the pelvis) and rectus abdominis (lumbar lordosis control). In some patients, it was possible to progress and vary the third posture in the sitting position, but this position was tiring for many patients and difficult to maintain properly (Figure 1).

At the end of the 10 Mézières treatment sessions, the patients kept their normal activities and then were assessed again in the 12-week follow-up.

For ethical reasons, we did not consider a third group in the waiting list without any rehabilitation treatment.

### 2.5. Home Exercise Group

The home exercises consisted of simple exercises that were performed by the patients at home, accompanied by spontaneous or diaphragmatic breathing, if necessary, based on the PD guidelines [32, 33].

While performing each exercise, the patient was allowed to use a support if he felt insecure. No exercise had to exacerbate or cause pain during its execution. The exercise program progressed in difficulty. The treatment was administered over 10 sessions, twice per week for 5 weeks. Each session lasted for 1 hour. The patients kept their normal activities and then were assessed again in the 12-week follow-up.

First, each patient attended two single 1-hour educational sessions with the physical therapist to learn how to perform the exercises at home well. Each patient was contacted by telephone every 2 weeks to monitor his/her adherence to the rehabilitation program. A booklet with an explanation and pictures of the exercises was given to the patients: each exercise was proposed for 3 sets of 10 repetitions with a rest period of at least 2 minutes between sets.

- *From Weeks 1 to 2*. Supine position: (i) spontaneous and diaphragmatic breathing, (ii) rolling on the side, (iii) bridge exercise, by lifting the legs alternately, (iv) prone position: exercise of the greeting from a crouching position, (v) in quadruped position, pelvic tilt exercises, and (vi) in crawling position for cross-pattern exercise.
- *From Weeks 2 to 3 (Add Exercises with respect to the First Few Weeks)*. (vii) In the cavalier servant position: moving the legs alternately, (viii) in sitting position: hands-knees cross-pattern, (ix) upright: legs slightly apart, semilateral squats, and (x) sitting position: rotation of the trunk to the right and left.
- *From Weeks 3 to 5 (Add Exercises)*. (xi) From sitting to standing position without support: postural step exercises, (xii) upright, semifront squats, moving the legs alternately, (xiii) upright position: back semisquat exercises, moving the legs alternately, (xiv) upright, semisquat exercises with the trunk leaning against the wall and interposition of a soft ball, both legs together, and (xv) upright, exercises with a stick for rotation and flexion-extension movements of the shoulder.

In the treatment program, the number of repetitions was allowed to increase. When possible, it was recommended that the exercises be performed in open spaces. Each rehabilitation session was preceded by a preparation phase of 15 minutes of low-intensity aerobic impact exercises (as walking) with adequate rest phases if necessary.

### 2.6. Sample Size Calculation

Given that there are no similar studies in the literature that used the Mézières method in PD, data from our preliminary pilot study of 10 patients with PD, considering the same inclusion and exclusion criteria previously described in the Materials and Methods, was used to determine the sample size, based on the following assumptions: (i) average risk of falling in patients with Parkinson's disease > 40 on the Berg Balance Scale and specifically a medium score of 45 for BBS with a standard deviation (SD) = 4 and (ii) an increase of 4 points after Mézières treatment on the Berg Balance Scale for minimal clinically important change per the literature [34]. A significance level of 95% was considered for a power of 80% by two-tailed *t*-test. The sample size was increased by 10% to account for eventual dropouts. The number of patients enrolled in each group was thus 17 (http://www.statisticalsolutions.net/pssTtest_calc.php). We did not publish the data of this preliminary study.

### 2.7. Data Analysis

A nonparametric approach was used, based on the low number of patients and assessing the normality using Shapiro-Wilk's test. The descriptive statistics were expressed as median with interquartile range (IQR) for quantitative variables according to the nonparametric approach and as percentage and tables of frequencies for qualitative ones. To compare the control versus treatment groups at the 3 times (*T*0,* T*1, and* T*2), nonparametric Mann–Whitney test was performed. To determine the significance difference in each group between* T*0,* T*1, and* T*2, we applied the nonparametric Friedman test for intragroup assessment and Wilcoxon test with Bonferroni correction (0.017 is the critical level of significance of Bonferroni correction, i.e., 0.05/3). The effect size for the post hoc significant comparisons was calculated. The chi-squared test was used to determine whether there is a significant difference between the categorical variables. If the expected counts were below 5, Fisher's Exact Test was applied as an alternative to a chi-square test for 2 × 2 tables.

SPSS version 20.0 (Chicago, IL, USA) was used for the statistical analyses. All tests were two-tailed with a level of significance of *p* < .05.

## 3. Results

### 3.1. Sample Characteristics

**Figure 2:**
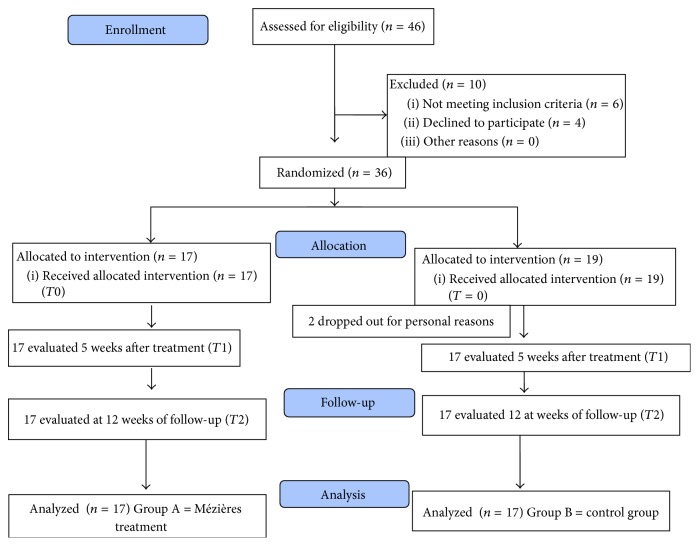
CONSORT flow diagram.

Forty-six patients were assessed for eligibility (*N* = 46), of whom 10 were excluded for not meeting the inclusion criteria (*N* = 6) or refusal to participate (*N* = 4). Ultimately, 36 patients (*N* = 36) were enrolled and randomly assigned into 2 groups: *n* = 17 in the Mézières group (Group A) and *n* = 19 in the control group (Group B). Two subjects left the control group during the treatment between* T*0 and* T*1 due to family issues; their data were not included in the statistical analysis because the evaluation scales had not been completed in whole parts (flowchart, Figure 2). Thus, the data for 17 patients per group were analyzed.

Baseline scores (*T*0) did not differ significantly with regard to age, BMI, gender, or evaluation scale scores, except for UPDRS Total. The patient characteristics at baseline are listed in Table 1, with a median age of 66 years (IQR = 18.50) for Group A and 67 (IQR = 11.00) for Group B; the median UPDRS Total scores were 44 (IQR: 0.00) and 44 (IQR: 7.50), respectively (*p* = .04). The mean of the duration of the disease was 3 ± 1.2 years with a Hoehn and Yahr score of 1.5 ± 0.8 for all the sample without statistically significant differences if we consider the two groups separately.

### 3.2. Between Groups

**Figure 3:**
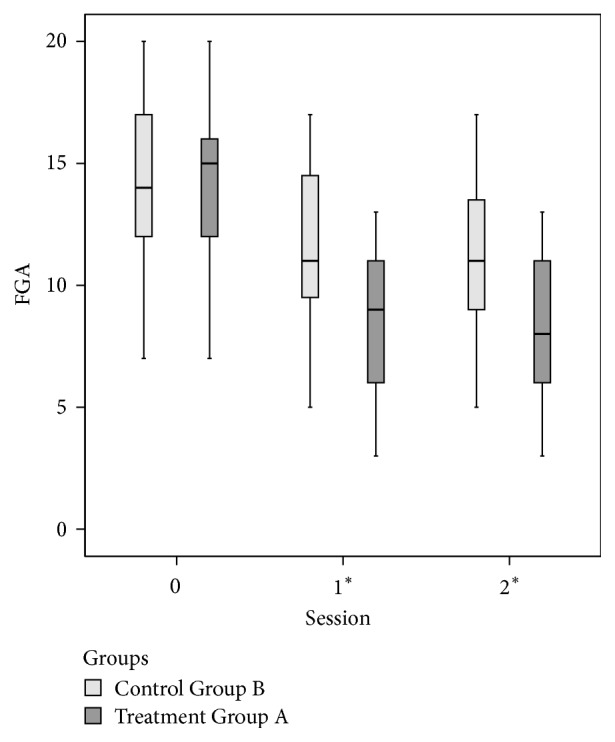
Functional Gait Assessment (FGA) at baseline (=*T*0) and at the end of treatment (=*T*1) and follow-up (=*T*2) for the two groups. The symbol “*∗*” indicates *p* < .05.

**Figure 4:**
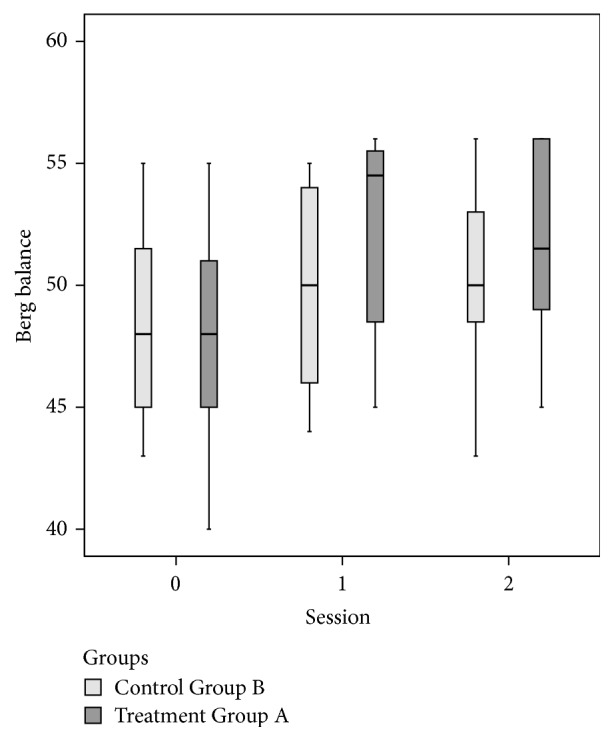
Berg Balance Scale (BBS) at baseline (=*T*0) and at the end of the treatment (=*T*1) and follow-up (=*T*2) for the two groups.

**Figure 5:**
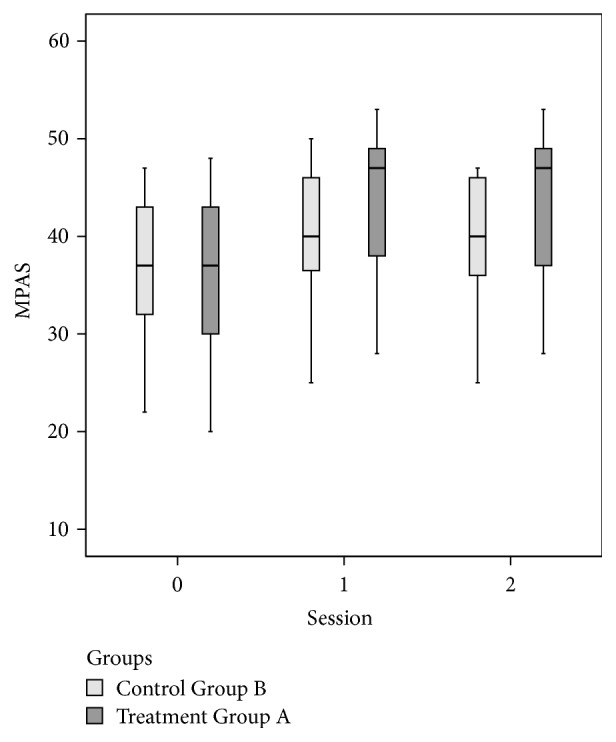
Modified Parkinson's Activity Scale (MPAS) at baseline (=*T*0) and at the end of the treatment (=*T*1) and follow-up (=*T*2) for the two groups.

By Mann–Whitney test, there were nonsignificant differences between Groups A and B except for FGA (*p* = .027 at *T*1 and *p* = .03 at *T*2) and for UPDRS Total (*p* = .007 at *T*1) (Figures 3, 4, and 5 and Tables 2 and 3).

### 3.3. In the Mézières Group

By Wilcoxon signed-rank test with Bonferroni correction, we observed significant changes in the Mézières-treated group between *T*0 and *T*1 for VAS (*p* < .001, *p* = .004), RP-SF36 (*p* = .019), Berg Balance Scale (*p* = .004), trunk flexion test (*p* < .001), FGA (*p* < .001), SMWT (*p* = .002), MPAS (*p* < .001), and UPDRS Total (*p* < .001) (UPDRS Part I, *p* = <.001; Part II, *p* = .001; and Part III, *p* ≤ .001). In addition, these statistically significant results were maintained at the follow-up (*T*2) except for RP-SF36 (Table 4).

### 3.4. In the Control Group

In the control group (Table 5), we noted significant changes between *T*0 and *T*1 for trunk flexion test (*p* = .013), FGA (*p* = .001), SMWT (*p* = .012), MPAS (*p* = .001), and UPDRS Total (*p* < .001) (UPDRS Part II, *p* = .028; Part IV, *p* = .010). At the follow-up (*T*0 versus *T*2), the results were significant for FGA, MPAS, and UPDRS Total (Part IV) and trunk flexibility. No adverse events or side effects in each intervention group were observed.

## 4. Discussion

The primary aim of our study was to determine the efficacy of the Mézières method in improving trunk flexibility of the back muscles and balance in patients with Parkinson's disease (PD). With regard to the risk of falls per the BBS and dynamic balance per the FGA, the Mézières approach resulted to be effective as the control group rehabilitative program. In particular, the Mézières treatment effected greater improvements on the BBS, which were stable at the follow-up, versus the control group. Even in the early stages of PD, alignment of the spine curves in the sagittal plane is lost, and the perception [31] of the body midline [32] adversely affects postural control, increasing postural instability and the risk of falls.

Some authors proposed that proprioceptive training, based on the association of many intensive perceptive stimuli during cognitive tasks that are focused on improving proprioception and sensory integration, helps patients with PD restore correct midline perception and, in turn, improves postural control in realigning the body midline to the gravitational axis [33].

The Mézières approach focuses on “awareness” of the trunk, alignment of the sagittal curves of the column, and alignment of the trunk, even with respect to the midline of the body. Thus, proprioception rehabilitation [3] approaches that solely target kinesthetic awareness [32] are recommended for patients with PD.

The Mézières method appears to synthesize both of these rehabilitative aspects to improve the kinesthetic and proprioceptive awareness of the trunk. When a patient adopts his posture during the progression of the Mézières regimen, the physical therapist always asks him to feel the stretch and recognize the position of the body and focus on the tactile sensations of the body surface. In our research, we have directed Mézières realignment towards the stretching of the latissimus dorsi, which is considered primarily to be a muscle with actions at the shoulder but also potentially makes contributions to lumbar spine function. Other data have demonstrated how this muscle affects spine-stabilizing ability to generate force and change length throughout the spine and ranges of motion in the shoulder [34].

Jobst et al. reported that patients learn to generate internal adaptive strategies with a combination of active posture correction strategies [35]; thus, working on the representation of the midline and motor imagery of the trunk to bridge perception and movement can yield new functional strategies for patients with PD to generate an internal cue reference. Further, in our study, dynamic balance and gait, as assessed with the FGA, improved significantly in the treatment group at* T*1 and* T*2.

Conversely, external cues might require less effort and attention by the patient, and their use during more complex activities could facilitate walking [36]. The Global Postural Reeducation (GPR), as a physical therapy approach that is based on the stretching of antigravity muscle chains with parallel enhancement of the basal tone of antagonistic muscles, improves the kinematic gait pattern, as evidenced by the recovery of the flexion amplitude of the knee and thigh [37]. Further, in the Mézières group, we observed significant differences in FGA, with good improvement in walking balance activity.

A secondary goal of our study was to determine the impact of Mézières rehabilitation exercises on pain, but we found no significant differences between the groups at the three observation times; thus, both approaches were efficacious with respect to pain relief.

Chronic pain, a distressing nonmotor symptom that is experienced by up to 85% of people with PD, is correlated with disease-related factors, such as rigidity, and daily living activities, such as coexisting musculoskeletal and neuropathic pain conditions. Moreover, exercise can activate the dopaminergic and nondopaminergic pain inhibitory pathways, suggesting that exercise helps modulate the experience of pain in PD [38]. Thus, the Mézières approach, through generating awareness of the body, could introduce the patient to a new experience without pain through exercise.

Chronic pain can negatively affect the HRQoL of patients with PD [17], necessitating additional rehabilitation focused on non-motor-associated pain. In our study, the control group did not experience any improvement in the quality of life, whereas in the Mézières group, RP and VT scores improved, Thus, we hypothesize that the presence of a physical therapist who guided the session has a positive impact on the perception of quality of life, leading the patient to feel more cared for and with a higher takeover of the patient together with the improvement in physical functioning and improvement in UPDRS.

Another important factor in the treatment group was the sequence of exercises. We first proposed a more postural and global approach to the body, closely related to control of the body midline and the recovery of the extensibility of the hypertonic muscle groups, in particular those of the low back muscular chain, such as the latissimus dorsi. Then, the subsequent steps were based on more dynamic exercises to ensure patient safety and autonomy in his motor habits.

Exercising consistently and beginning regular exercise were associated with small but significant positive effects on HRQoL and mobility over 2 years [39].

*Strengths*. This study was designed as a randomized controlled trial, which is considered the ideal methodological approach for evaluating the efficacy of a specific intervention. This study is the first trial to assess the efficacy of the Mézières approach in improving balance stability and trunk flexibility in PD.

*Limits*. The findings might be applicable only to patients who experience mild-to-moderate symptoms and are healthy enough to perform the exercises. Thus, alternative interventions might be necessary for patients who present with more advanced symptoms. All evaluations and rehabilitation treatments were performed during the “on” phase.

## 5. Conclusions

Outcomes of the present study suggest that the Mézières approach is efficacious in improving balance in patients with PD and is also a good exercise program with a focus on increasing flexibility in the stronger muscles; strengthening of back muscles can help keep the spine erect in PD patients. It would be desirable in the future to include the Mézières method in multidisciplinary rehabilitation protocols for patients with Parkinson's disease with longer rehabilitation sessions.

## Figures and Tables

**Table 1:** Characteristics of the groups with regard to gender, age, and BMI (body mass index) at baseline (median and IQR, frequencies, and percentages).

Characteristics	Control group (*N* = 17)		Mézières group (*N* = 17)		
*Qualitative variables*	*N*	%	*N*	%	*p*
Gender					
Female	8	47	7	41	
Male	9	53	10	59	.95

*Continuous variables*	*Median*	*IQR*	*Median*	*IQR*	*p*
Age	67.00	11.00	66.00	18.50	.65
BMI	25.00	5.50	25.80	2.90	.99

IQR: interquartile range; BMI: body mass index.

**Table 2:** Quality of life: comparison between groups at *T*1 (end of treatment) and *T*2 (follow-up) for SF-36 and subscales.

SF-36	Group B (*N* = 17) *T*1		Group A (*N* = 17) *T*1		*p* ^*∗*^	Group B (*N* = 17) *T*2		Group A (*N* = 17) *T*2		*p* ^*∗*^
	Median	IQR	Median	IQR		Median	IQR	Median	IQR	
PF	75.00	35.00	75.00	15.00	.40	85.00	45.00	85.00	25.00	.90
PR	100.00	50.00	100.00	50.00	.50	100.00	45.00	100.00	37.50	.60
BD	61.00	31.00	62.00	30.00	.20	61.00	50.00	72.00	43.50	.20
GH	47.00	25.00	47.00	24.80	.60	45.00	33.00	42.00	37.50	.90
VT	50.00	15.00	57.50	15.00	.10	50.00	23.00	55.00	27.50	.50
SF	75.00	25.00	87.30	25.00	.60	87.50	23.00	87.50	25.00	.75
RE	66.70	34.00	100.00	33.80	.20	100.00	50.00	100.00	33.40	.40
MH	64.00	28.00	70.00	28.00	.30	60.00	28.00	76.00	22.00	.10

^*∗*^ *p* value by Mann–Whitney test. Group A: Mézières treatment group; Group B: control group. IQR: interquartile range; SF-36: Short Form 36 Health Survey; PF: physical functioning; PR: physical role functioning; BD: bodily pain; GH: general health perceptions; VT: vitality; SF: social role functioning; RE: emotional role functioning; MH: mental health.

**Table 3:** Scales and clinical evaluation: comparison between groups at *T*1 (end of treatment) and *T*2 (follow-up) for pain, mobility (balance and posture), and disability (median and IQR, *p* < .05).

Scales and clinical evaluations	Group B (*N* = 17) *T*1		Group A (*N* = 17) *T*1		*p* ^*∗*^	Group B (*N* = 17) *T*2		Group A (*N* = 17) *T*2		*p* ^*∗*^
	Median	IQR	Median	IQR		Median	IQR	Median	IQR	
VAS (cm)	2.50	4.00	2.00	4.00	.20	3.00	4.00	1.50	4.00	.80

BBS	50.00	8.00	54.50	8.00	.10	50.00	5.00	51.50	7.50	.28

Trunk flexion test (cm)	10,00	10.00	10.00	11.80	.60	10.00	10.00	8.00	12.00	.50

FGA	11.00	6.00	9.00	6.00	**.027**	11.00	5.00	8.00	6.00	**.03**

MPAS	40.00	10.00	47.00	13.50	.30	40.00	10.00	47.00	14.00	.11

SMWT (min)	500.00	145.00	510.00	171.30	.90	500.00	130.00	480.00	277.50	.80

UPDRS Part I	7.00	7.00	7.50	7.00	.3	8.00	8.00	5.00	7.00	.05
UPDRS Part II	7.00	6.00	6.00	5.80	.40	6.00	8.00	6.00	5.50	.90
UPDRS Part III	11.00	8.00	10.00	8.50	.50	13.00	10.00	10.00	9.00	.20
UPDRS Part IV	2.00	5.00	2.50	4.80	.98	0.99	5.00	3.00	4.50	.95
UPDRS Total	49.00	0.00	49.00	12.50	**.007**	37.00	0.00	38.00	20.00	.80

^*∗*^ *p* value by Mann–Whitney test. Group A: Mézières treatment group; Group B: control group. IQR: interquartile range. VAS: Visual Analog Scale; BBS: Berg Balance Scale; FGA: Functional Gait Assessment; MPAS: Modified Parkinson's Activity Scale; SMWT: six-minute walking test; UPDRS: Unified Parkinson's Disease Rating Scale.

**Table 4:** Scales and clinical evaluation: comparison for Mézières group at 3 evaluation times.

Scales and clinical evaluations for Mézières group	*T*0		*T*1		*T*2		*p* ^a^	WB^b^	Effect size^c^
	Median	IQR	Median	IQR	Median	IQR			
VAS (cm)	3.0	4.0	2.0	4.0	1.5	4.0	**<0.001**	1; 2	0.24; 0.25

SF-36 PF	72.5	33.8	75.0	15.0	85.0	25.0	0.014	—	
SF-36 RP	75.0	72.5	100.0	50.0	100.0	37.5	**0.002**	1	0.24
SF-36 BP	62.0	22.3	62.0	30.0	72.0	43.5	0.598	—	
SF-36 GH	42.0	29.0	47.0	25.0	42.0	37.5	0.198	—	
SF-36 VT	50.0	15.0	57.5	15.0	55.0	27.5	0.018	—	
SF-36 SF	75.0	34.8	87.3	25.0	87.5	25.0	**0.002**	—	
SF-36 RE	100.0	66.7	100.0	33.8	100.0	33.0	0.368	—	

BBS	48.0	6.5	54.5	8.0	51.5	7.5	**<0.001**	1; 2	0.25; 0.25

Trunk flexion test (cm)	10.5	10.0	10.0	11.8	8.0	12.0	**<0.001**	1; 2	0.24; 0.24

FGA	15.0	5.0	9.0	6.0	8.0	6.0	**<0.001**	1; 2	0.24; 0.24

MPAS	37.0	16.0	47.0	13.5	47.0	14.0	**<0.001**	1; 2	0.24; 0.24

SMWT (min)	467.5	157.5	510.0	171.3	480.0	277.5	0.081	—	

UPDRS Part I	10.0	9.0	7.5	7.0	5.0	7.0	**<0.001**	1; 2	0.24; 0.24
UPDRS Part II	9.0	6.8	6.0	5.8	6.0	5.5	**<0.001**	1; 2	0.24; 0.24
UPDRS Part III	15.5	10.8	10.0	8.5	10.0	9.0	**<0.001**	1; 2	0.24; 0.24
UPDRS Total	38.0	20	27.0	12.5	23.0	16.5	**<0.001**	1; 2	0.24; 0.24

Group A: Mézières treatment group; IQR: interquartile range; VAS: Visual Analog Scale; SF-36: Short Form 36 Health Survey; PF: physical functioning; PR: physical role functioning; BD: bodily pain; GH: general health perceptions; VT: vitality; SF: social role functioning; RE: emotional role functioning; MH: mental health; BBS: Berg Balance Scale; FGA: Functional Gait Assessment; MPAS: Modified Parkinson's Activity Scale; SMWT: six-minute walking test; UPDRS: Unified Parkinson's Disease Rating Scale. ^a^*p* value obtained by Friedman test. ^b^Significant comparisons obtained by Wilcoxon test with Bonferroni correction (0.017 is the critical level of significance of Bonferroni correction, i.e., 0.05/3: 1 → *T*0 versus *T*1; 2 → *T*0 versus *T*2; 3 → *T*1 versus *T*2; not significant comparisons). ^c^Effect size for the post hoc comparison. Bold font indicates statistical significance.

**Table 5:** Scales and clinical evaluation: comparison for the control group at 3 evaluation times.

Scales and clinical evaluations for the control group	*T*0		*T*1		*T*2		*p* ^a^	WB^b^	Effect size^c^
	Median	IQR	Median	IQR	Median	IQR			
VAS (cm)	3.0	3.5	2.5	4.0	3.0	4.0	0.226	—	

SF36-PF	75.0	45.0	75.0	35.0	85.0	45.0	0.108	—	
SF36-RP	50.0	75.0	100.0	50.00	100.0	45.0	0.054	—	
SF-36 BP	61.0	20.0	61.0	31.00	61.0	50.0	0.497	—	
SF-36 GH	42.0	13.0	47.0	25.00	45.0	33.0	0.627	—	
SF-36 VT	50.0	10.0	50.0	15.00	50.0	23.0	0.412	—	
SF-36 SF	75.0	38.0	75.0	25.00	87.5	23.3	0.575	—	
SF-36 RE	66.7	66.7	66.7	34.00	100.0	50.0	0.430	—	
SF-36 MH	68.0	16.0	64.0	28.00	60.0	28.0			

BBS	48.00	7.0	50.0	8.00	50.0	5.0	0.360	—	

Trunk flexion test (cm)	11.00	10.0	10.0	10.00	10.0	10.0	0.012	1; 2	0.26; 0.26

FGA	14.00	6.0	11.0	6.00	11.0		**<0.001**	1; 2	0.26; 0.26

MPAS	37.00	13.0	40.0	10.00	40.0	10.0	**<0.001**	1; 2	0.26; 0.26

SMWT (min)	480.00	120.0	500.0	145.00	500.0	130.0	**0.005**	1	0.26

UPDRS Part I	10.00	8.0	7.0	7.00	8.0	8.0	0.262	—	
UPDRS Part II	9.00	9.0	7.0	6.00	6.0	8.0	0.050	—	
UPDRS Part III	14.00	10.0	11.0	8.00	13.0	10.0	0.064	—	
UPDRS Part IV	4.00	6.0	2.0	5.00	0.9	5.0	**<0.001**	1; 2	0.26; 0.26
UPDRS Total	44.00	0.0	49.0	0.00	37.0	0.0	**0.015**	2	

Group B: control group; IQR: interquartile range; SF-36: Short Form 36 Health Survey; PF: physical functioning; PR: physical role functioning; BD: bodily pain; GH: general health perceptions; VT: vitality; SF: social role functioning; RE: emotional role functioning; MH: mental health; VAS: Visual Analog Scale; BBS: Berg Balance Scale; FGA: Functional Gait Assessment; MPAS: Modified Parkinson's Activity Scale; SMWT: six-minute walking test; UPDRS: Unified Parkinson's Disease Rating Scale. ^a^*p* value obtained by Friedman test. ^b^Significant comparisons obtained by Wilcoxon test with Bonferroni correction (0.017 is the critical level of significance of Bonferroni correction, i.e., 0.05/3: 1 → *T*0 versus *T*1; 2 → *T*0 versus *T*2; 3 → *T*1 versus *T*2; not significant comparisons). ^c^Effect size for the post hoc comparison. Bold font indicates statistical significance.

## References

[1] Mézières F. (1949). *La Révolution en Gymnastique Orthopédique*.

[2] Coelho L. O. Anti-fitness ou o Manifesto Anti-Desportivo.

[3] Mézières F. (1984). *Originalité de la Méthode Mézières*.

[4] Melamed E., Djaldetti R. (2006). Camptocormia in Parkinson's disease. *Journal of Neurology*.

[5] Wunderlich S., Csoti I., Reiners K. (2002). Camptocormia in Parkinson's disease mimicked by focal myositis of the paraspinal muscles. *Movement Disorders*.

[6] Curtze C., Nutt J. G., Carlson-Kuhta P., Mancini M., Horak F. B. (2016). Objective gait and balance impairments relate to balance confidence and perceived mobility in people with parkinson disease. *Physical Therapy in Sport*.

[7] Mathevon L., Leroux N., Piscicelli C. (2016). Sustainable reduction in the occurrence of falls in a Parkinson's patient who followed an intensive and specific rehabilitation program to recalibrate verticality perception. *Annals of Physical and Rehabilitation Medicine*.

[8] Paolucci T., Morone G., Fusco A. (2014). Effects of perceptive rehabilitation on balance control in patients with Parkinson's disease. *NeuroRehabilitation*.

[9] Leavy B., Roaldsen K. S., Nylund K., Hagströmer M., Franzén E. (2017). “Pushing the limits”: Rethinking motor and cognitive resources after a highly challenging balance training program for parkinson disease. *Physical Therapy in Sport*.

[10] Doná F., Aquino C. C., Gazzola J. M. (2016). Changes in postural control in patients with Parkinson's disease: a posturographic study. *Physiotherapy (United Kingdom)*.

[11] Schlenstedt C., Paschen S., Kruse A., Raethjen J., Weisser B., Deuschl G. (2015). Resistance versus balance training to improve postural control in Parkinson’s disease: a randomized rater blinded controlled study. *PLoS ONE*.

[12] Alves Da Rocha P., McClelland J., Morris M. E. (2015). Complementary physical therapies for movement disorders in parkinson’s disease: a systematic review. *European Journal of Physical and Rehabilitation Medicine*.

[13] Abbruzzese G., Marchese R., Avanzino L., Pelosin E. (2016). Rehabilitation for Parkinson's disease: current outlook and future challenges. *Parkinsonism & Related Disorders*.

[14] Fil A., Cano-de-la-Cuerda R., Muñoz-Hellín E., Vela L., Ramiro-González M., Fernández-de-las-Peñas C. (2013). Pain in Parkinson disease: a review of the literature. *Parkinsonism & Related Disorders*.

[15] Wallace V. C. J., Chaudhuri K. R. (2014). Unexplained lower limb pain in Parkinson's disease: A phenotypic variant of "painful Parkinson's disease". *Parkinsonism & Related Disorders*.

[16] Lawand P., Lombardi Júnior I., Jones A., Sardim C., Ribeiro L. H., Natour J. (2015). Effect of a muscle stretching program using the global postural reeducation method for patients with chronic low back pain: A randomized controlled trial. *Joint Bone Spine*.

[17] Capecci M., Serpicelli C., Fiorentini L. (2014). Postural rehabilitation and kinesio taping for axial postural disorders in Parkinson's disease. *Archives of Physical Medicine and Rehabilitation*.

[18] Agosti V., Vitale C., Avella D. (2016). Effects of Global Postural Reeducation on gait kinematics in parkinsonian patients: a pilot randomized three-dimensional motion analysis study. *Neurological Sciences*.

[19] Hoehn M. M., Yahr M. D. (1967). Parkinsonism: onset, progression and mortality.. *Neurology*.

[20] Folstein M. F., Folstein S. E., McHugh P. R. (1975). “Mini mental state”. A practical method for grading the cognitive state of patients for the clinician. *Journal of Psychiatric Research*.

[21] Ferrazzoli D., Ortelli P., Maestri R. (2016). Does cognitive impairment affect rehabilitation outcome in Parkinson's disease?. *Frontiers in Aging Neuroscience*.

[22] Moher D., Hopewell S., Schulz K. F. (2012). CONSORT 2010 explanation and elaboration: updated guidelines for reporting parallel group randomised trials. *International Journal of Surgery*.

[23] Berg K. O., Wood-Dauphinee S. L., Williams J. L. (1992). Measuring balance in the elderly: validation of an instrument. *Canadian Journal of Public Health*.

[24] Huskisson E. C. (1974). Measurement of pain. *The Lancet*.

[25] Wrisley D. M., Marchetti G. F., Kuharsky D. K., Whitney S. L. (2004). Reliability, internal consistency, and validity of data obtained with the functional gait assessment. *Physical Therapy in Sport*.

[26] Nieuwboer A., De Weerdt W., Dom R., Bogaerts K., Nuyens G. (2000). Development of an activity scale for individuals with advanced Parkinson disease: Reliability and 'on-off' variability. *Physical Therapy in Sport*.

[27] Keus S. H. J., Nieuwboer A., Bloem B. R., Borm G. F., Munneke M. (2009). Clinimetric analyses of the Modified Parkinson Activity Scale. *Parkinsonism & Related Disorders*.

[28] Fahn S., Elton R. L., Members of the UPDRS Development Committee, Fahn S., Marsden C. D., Calne D. B., Goldstein M. (1987). Unified Parkinson’s disease rating scale. *Recent Developments in Parkinson’s Disease*.

[29] Butland R. J. A., Pang J., Gross E. R., Woodcock A. A., Geddes D. M. (1982). Two-, six-, and 12-minute walking tests in respiratory disease. *British Medical Journal*.

[30] Ware J. E. (2000). SF-36 health survey update. *The Spine Journal*.

[31] Apolone G., Mosconi P. (1998). The Italian SF-36 Health Survey: translation, validation and norming. *Journal of Clinical Epidemiology*.

[32] Weiner W. J. (2002). An algorithm (decision tree) for the management of Parkinson's disease (2001): treatment guidelines. *Neurology*.

[33] Keus S., Munneke M., Graziano M. European Physiotherapy Guideline for Parkinson's Disease.

[34] Donoghue D., Stokes E. K. (2009). How much change is true change? The minimum detectable change of the Berg Balance Scale in elderly people. *Journal of Rehabilitation Medicine*.

[35] Jobst E. E., Melnick M. E., Byl N. N., Dowling G. A., Aminoff M. J. (1997). Sensory perception in Parkinson disease. *JAMA Neurology*.

[36] Manzoni T., Barbaresi P., Conti F., Fabri M. (1989). The callosal connections of the primary somatosensory cortex and the neural bases of midline fusion. *Experimental Brain Research*.

[37] Morrone M., Miccinilli S., Bravi M. (2016). Perceptive rehabilitation and trunk posture alignment in patients with Parkinson disease: a single blind randomized controlled trial. *European Journal of Physical and Rehabilitation Medicine*.

[38] Gerling M. E., Brown S. H. M. (2013). Architectural analysis and predicted functional capability of the human latissimus dorsi muscle. *Journal of Anatomy*.

[39] Ceravolo M. G. (2009). Rehabilitation goals and strategies in Parkinson's disease. *European Journal of Physical and Rehabilitation Medicine*.

